# Avulsion fracture of olecranon treated with McLaughlin-cerclage using an artificial ligament: a case report

**DOI:** 10.1093/jscr/rjae542

**Published:** 2024-08-28

**Authors:** Yusei Katsuyama, Shinichiro Nakamura, Kentaro Sasaki, Koichi Umeda, Kenji Takahashi

**Affiliations:** Department of Orthopedics, Graduate School of Medical Science, Kyoto Prefectural University of Medicine, 465 Kajii-cho, Kamigyo-ku, Kyoto 602-8566, Japan; Department of Orthopaedics, Fukuchiyama City Hospital, 231 Atsunaka-cho, Fukuchiyama, Kyoto 620-8505, Japan; Department of Orthopaedics, Fukuchiyama City Hospital, 231 Atsunaka-cho, Fukuchiyama, Kyoto 620-8505, Japan; Department of Orthopaedics, Fukuchiyama City Hospital, 231 Atsunaka-cho, Fukuchiyama, Kyoto 620-8505, Japan; Department of Orthopedics, Graduate School of Medical Science, Kyoto Prefectural University of Medicine, 465 Kajii-cho, Kamigyo-ku, Kyoto 602-8566, Japan

**Keywords:** olecranon fracture, avulsion fracture, McLaughlin cerclage, artificial ligament, osteopenia

## Abstract

A 65-year-old woman presented with right elbow pain after a fall. Imaging showed an avulsion fracture of the olecranon. The patient subsequently underwent surgery using the suture bridge technique with anchors. However, loosening was observed intraoperatively. Therefore, a McLaughlin cerclage with an artificial ligament was added, resulting in a rigid fixation. Bone union was achieved at 4 months postoperatively. At 18 months postoperatively, no limitation was observed in the range of motion of the elbow joint; the disabilities of the arm, shoulder and hand score was 0. McLaughlin cerclage with an artificial ligament provided additional fixation, demonstrating greater strength compared with suture anchors and minimizing the risk of cut-through in the osteoporotic bone. This approach offers a promising alternative for such cases by combining firm fixation with a reduced risk of complications, particularly in older patients with osteoporosis.

## Introduction

Olecranon fractures account for ~10% of elbow fractures [1]. Generally, fixation with tension-band wiring or plates is performed for olecranon fractures [2]. However, avulsion fractures of the olecranon cannot be strongly fixed using conventional methods because bone fragments are small. Furthermore, fixation is more challenging in patients with osteoporosis. Bone tunnel-based repair and suture anchor techniques have been reported for fractures [3, 4]. However, the optimal surgical treatment is controversial.

Thus, we aimed to contribute to the development of an optimal surgical strategy for olecranon fractures by presenting a case of an avulsion fracture in a patient with osteopenia treated with McLaughlin cerclage using an artificial ligament.

## Case report

**Figure 1:**
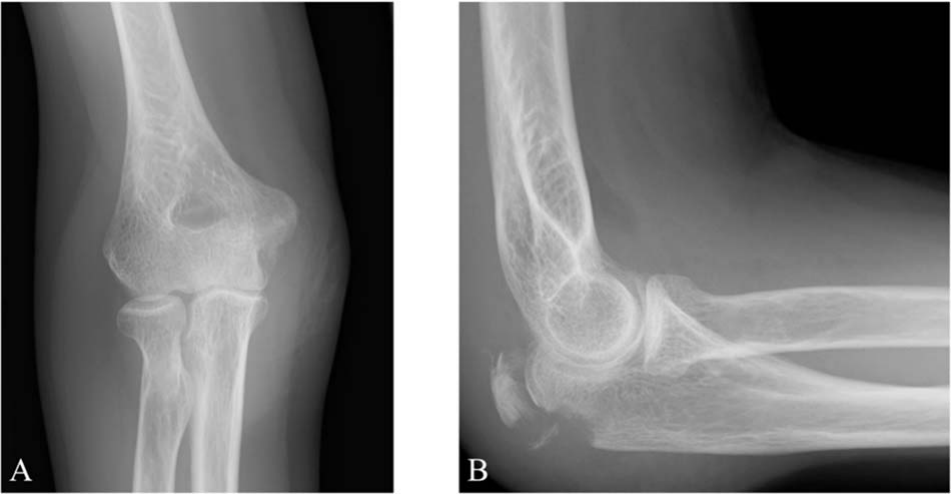
Plain radiographs of the right elbow show no abnormal findings in the anteroposterior view (A); however, an avulsion fracture of the olecranon is observed in the lateral view (B).

A 65-year-old healthy Japanese woman fell on her hands with her right elbow extended. She presented to our hospital with pain and swelling in the right elbow joint; active extension was impossible. X-rays showed a triceps tendon avulsion fracture (Fig. 1). Her bone mineral density, using dual X-ray absorptiometry, was 71% of the young adult mean for the lumbar spine. She was diagnosed with an avulsion fracture of the right olecranon (AO classification: 2U1A1) and underwent surgery 7 days post-injury.

**Figure 2:**
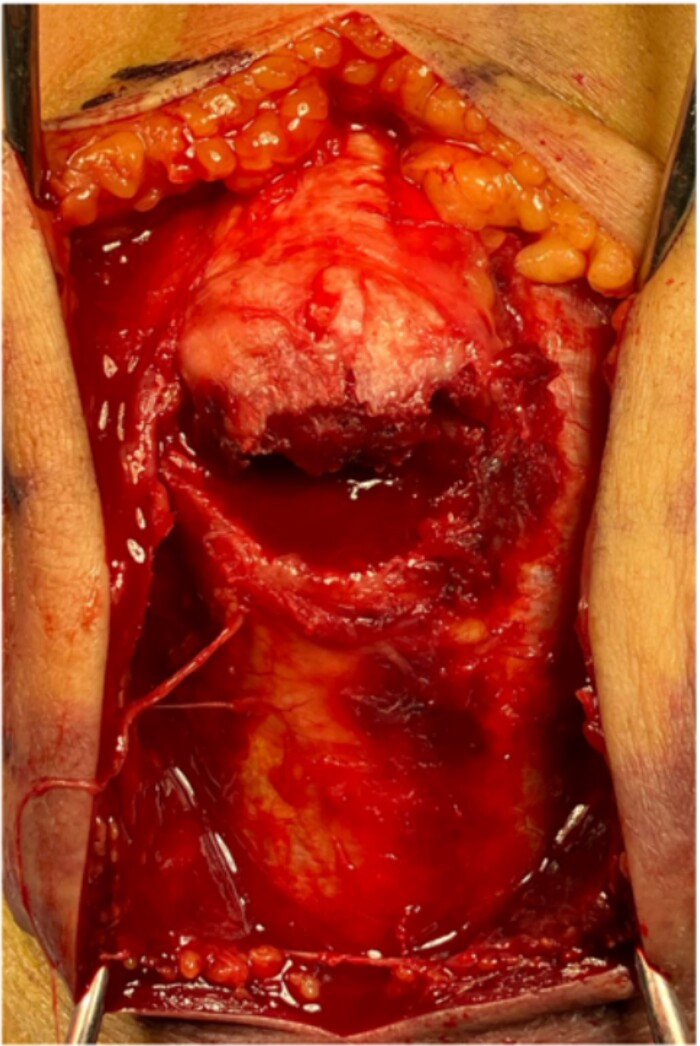
The bone fragment is displaced, and its continuity with the triceps tendon is preserved.

**Figure 3:**
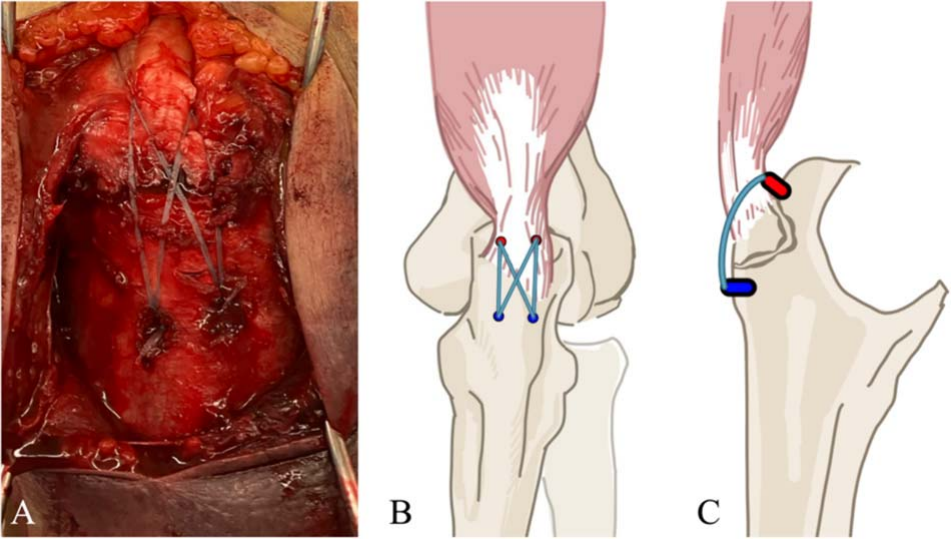
(A) The suture bridge technique is performed using JuggerKnot® All-Suture Anchors and Quattro® Link Knotless anchors. A schematic of the surgical field is shown in (B) frontal and (C) lateral views. Proximal anchors are JuggerKnot® All-Suture Anchors, while distal anchors are Quattro® Link Knotless anchors.

**Figure 4:**
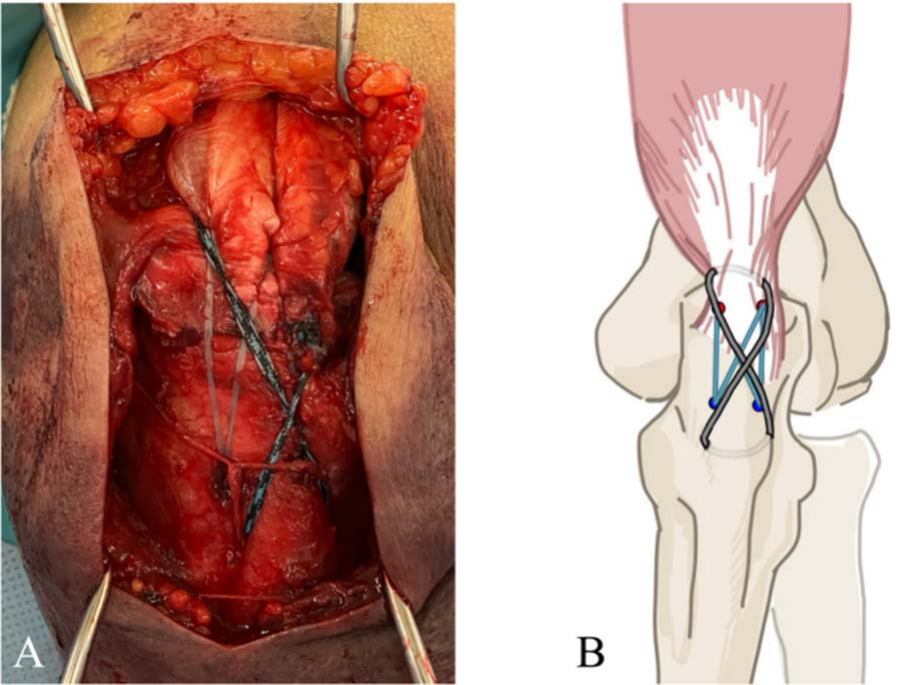
(A) Addition of McLaughlin cerclage using BroadBand® Tape. (B) A schematic of the surgical field is shown. The artificial ligament is fastened in a figure-of-eight configuration.

The surgery was performed in the left lateral recumbent position. An incision was made in the posterior midline over the right olecranon. Triceps brachii tendon rupture was not observed (Fig. 2). The fracture fragment was inverted, and two 1.4-mm JuggerKnot® All-Suture Anchors (Zimmer Biomet, USA) were inserted into the ulna proximal to the fracture site without perforation into the elbow joint. Subsequently, sutures were threaded through the triceps brachii muscle; the dislocated bone fragment was pulled together with the triceps brachii muscle. Despite maintaining the elbow at a 30° flexion position, two Quattro® Link Knotless anchors (Zimmer Biomet, USA) were inserted into the ulna distal to the fracture site to reduce and fix the fragment using the suture bridge procedure (Fig. 3). However, upon flexing the elbow joint to 90°, the bone fragment was displaced and anchors were loose. Therefore, we opted to use the McLaughlin cerclage technique, utilizing a 2.3-mm BroadBand® Tape (Zimmer Biomet, USA) passing through a predrilled hole perpendicular to the ulnar shaft. The suture was crossed over the posterior aspect of the ulna and tightened in a figure-of-eight manner through the triceps brachii tendon (Fig. 4). This procedure resulted in no dislocation of the fracture site, even when the elbow joint was flexed to 130°.

**Figure 5:**
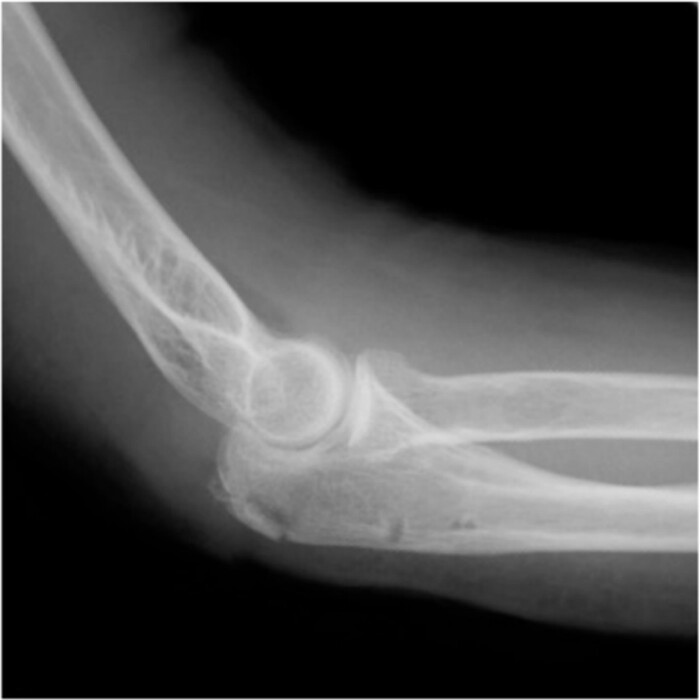
Postoperative lateral elbow radiographs. The bone fragment is reduced and fixed.

**Figure 6:**
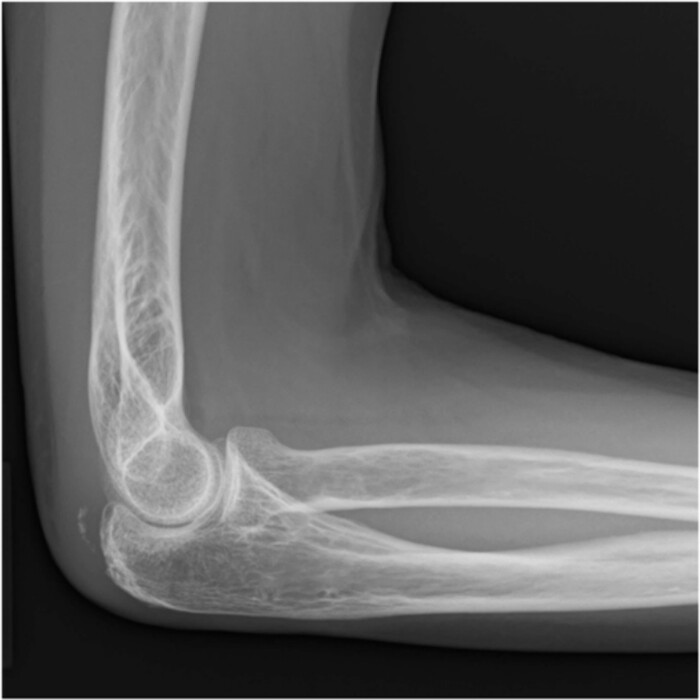
At 4 months after the surgery, the X-ray shows complete bone union in the lateral view.

Postoperative radiography revealed reduced bone fragmentation (Fig. 5). The elbow was immobilized at 90° for 1 week postoperatively; range-of-motion exercises were started in postoperative week 2. Radiographs obtained 4 months postoperatively showed bone union (Fig. 6). At 18 months postoperatively, the patient had a range of motion of 140° flexion and 0° extension. The disability of the arm, shoulder and hand score was 0.

## Discussion

Avulsion fractures of the olecranon are most common in men in their 40s and 50s and were primarily caused by direct contact and sports trauma [5]. Zacharia *et al.* [3] presented a classification and treatment algorithm for olecranon avulsion fractures. When a small bone fragment could not be fixed with tension-band wiring using Kirschner and cable wires, the triceps brachii tendon was sutured using the Krackow technique with high-strength sutures and passed through the tunnel drilled transversely to the ulna to secure it. This method resulted in favorable outcomes in all cases.

In contrast, reports on surgical techniques using suture anchors have increased in recent years. A biomechanical comparison showed that knotless double-row suture anchor repair exhibits strength comparable to that of transosseous cruciate distal triceps repair [6]. Furthermore, a systematic review of triceps ruptures reported that suture anchor repair had lower complication and retear rates compared with transosseous tunnel repair [4]. Furuhata *et al.* [7] reported about an avulsion fracture of the olecranon treated with the suture bridge technique. They highlighted three key features of the technique: no iatrogenic fracture risk during transcutaneous tunnel drilling; no postoperative knot failure or subcutaneous irritation; and blood flow maintenance to the suture site.

In our case, the suture bridge technique was initially performed, resulting in the loosening of the anchor. Although a literature review indicates the effectiveness of suture anchor repair, the repair effects in older women are unknown because >80% of patients enrolled in previous studies were male participants, with an average age on the younger side [4]. Biomechanical analysis has shown that the pull-through strength of an anchor is weaker in osteoporotic bone than that in normal bone [8]. Our patient, who was an older woman with osteopenic bone, is presumed to have experienced failure as a result of osteopenia.

We performed McLaughlin cerclage with an artificial ligament to enhance fixation. The McLaughlin cerclage was first reported in 1947 for treating patellar and Achilles tendon ruptures [9]. A biomechanical evaluation showed that McLaughlin cerclage offers a greater fixation strength than do suture anchors in cases of patellar tendon ruptures [10]. Furthermore, this study evaluated fixation materials and found no difference in strength between using polydioxanone sutures and cable wires, showing that strong fixation is achievable even with non-metallic materials. Murakami *et al.* [11] reported that polyethylene tape is wider than stainless steel and titanium cables, resulting in decreased local stress and increased resistance to cut-through. They suggested that wider wire could prevent cheese-cutting in the bone tunnels. In our case, we used artificial ligaments to achieve secure fixation while preventing cut-throughs. Artificial ligaments, being soft materials, cause less irritation and skin damage, thereby avoiding the need for removal. To our knowledge, our case is the first report of treatment of an avulsion fracture of the olecranon using the McLaughlin cerclage technique with an artificial ligament. This technique may provide an ideal method for firm fixation with reduced risk of complications.

In conclusion, we present a surgical procedure for treating olecranon avulsion using the McLaughlin cerclage technique with an artificial ligament. This procedure may be particularly beneficial in osteoporotic cases due to secure fixation and resistance to cut throughs.

## Data Availability

The datasets used and analysed in the current study are available from the corresponding author upon reasonable request.
